# Predisposition to Apoptosis in Hepatocellular Carcinoma: From Mechanistic Insights to Therapeutic Strategies

**DOI:** 10.3389/fonc.2019.01421

**Published:** 2019-12-13

**Authors:** Jens U. Marquardt, Frank Edlich

**Affiliations:** ^1^Department of Medicine I, University Medical Center Schleswig-Holstein, Lübeck, Germany; ^2^Department of Medicine, Lichtenberg Research Group, University Mainz, Mainz, Germany; ^3^Heisenberg Research Group “Regulation von Bcl-2-Proteinen Durch Konformationelle Flexibilität,” Institute for Biochemistry and Molecular Biology, University of Freiburg, Freiburg, Germany; ^4^CIBSS Centre for Integrative Biological Signalling Studies, University of Freiburg, Freiburg, Germany

**Keywords:** hepatocellular carcinoma, cell death, inflammation, BCL-2 family, BH3, primed to death, BH3 profiling, mitochondrial apoptosis

## Abstract

Hepatocellular carcinoma (HCC) ranks among the most rapidly evolving cancers in the Western world. The majority of HCCs develop on the basis of a chronic inflammatory liver damage that predisposes liver cancer development and leads to deregulation of multiple cellular signaling pathways. The resulting dysbalance between uncontrolled proliferation and impaired predisposition to cell death with consecutive failure to clear inflammatory damage is a key driver of malignant transformation. Therefore, resistance to death signaling accompanied by metabolic changes as well as failed immunological clearance of damaged pre-neoplastic hepatocytes are considered hallmarks of hepatocarcinogenesis. Hereby, the underlying liver disease, the type of liver damage and individual predisposition to apoptosis determines the natural course of the disease as well as the therapeutic response. Here, we will review common and individual aspects of cell death pathways in hepatocarcinogenesis with a particular emphasis on regulatory networks and key molecular alterations. We will further delineate the potential of targeting cell death-related signaling as a viable therapeutic strategy to improve the outcome of HCC patients.

## Introduction

The common hallmark of the vast majority of Hepatocellular carcinomas (HCC) is a chronic inflammatory liver damage induced by a diverse spectrum of etiological risk factors (1). Depending on the type of liver injury and persistence of the underlying inflammatory stimulus, HCCs are particularly characterized by a significant phenotypic and molecular heterogeneity. Therefore, HCCs are oncogenic paradigms for inflammation-induced cancers (2). Herein, the underlying causes of the chronic liver disease range from chronic hepatitis B (HBV) and C viruses (HCV) infections over excessive alcohol abuse to metabolic liver diseases. Importantly, the obesity-associated alterations of the hepatic microenvironment resembling non-alcoholic fatty liver disease and, more importantly, steatohepatitis (NAFLD/NASH) are now among the most prominent etiological risk factors for HCC in several Western countries (3). The particular type of inflammatory liver damage induced by NASH is also responsible for a high number of HCCs without underlying cirrhosis (4, 5). Given the rising incidence of the metabolic syndrome worldwide, it is not surprising that HCC currently ranks among the most rapidly evolving and deadliest cancers in the Western world. Further, the impaired liver function and observed molecular heterogeneity renders effective treatments of HCCs particularly challenging (6, 7).

In the context of HCC development and progression, special importance can be assigned to the type of liver damage and associated changes to the hepatic micromilieu that create a pro-oncogenic field effect and precede malignant transformation of hepatocytes (8, 9).

Various types of liver injury and associated chronic cell death responses have been identified to trigger inflammatory liver diseases, fibrosis development and, ultimately, hepatocarcinogenesis (10, 11). Accordingly, major cell death processes as well as signaling pathways are associated with liver cancer development and mainly involve apoptosis and necrosis. However, other forms of cell death, such as autophagy, necroptosis, pyroptosis, ferroptosis, or combinations of these death programs, have also been linked to HCC development and progression (11). Damaged hepatocytes induce activation and cross-talk of other non-parenchymal, immune and stromal cells with subsequent release of cytokine that fuel inflammation-induced damage and prone cancer development (12). Abnormalities in glucose and lipid metabolisms as well as microbiota composition further aggravate the oncogenic process. While the mentioned cell death mechanisms are relevant for hepatocarcinogenesis, regardless of the underlying etiological risk factors, oxidative stress and consecutive impairment of mitochondrial function seem to particularly induce hepatocyte death during metabolic liver damage and lead to signaling through B-cell lymphoma-2 (BCL-2) family proteins and activation of caspases and c-Jun N-terminal kinase during NASH-induced HCC (13). Besides prominent roles of cell death pathways in HCC development, cell death regulation and associated changes are also important for diagnosis and therapy. Several surrogate methods to assess and quantify liver injury, predominant mode of cell death and activation of inflammatory processes have been successfully evaluated in the context of acute and chronic liver diseases (14, 15). However, reliable and non-invasive cell death markers are not available in clinical routine. Cell death and inflammatory markers have also been assessed as prognostic markers or to facilitate monitoring of therapy response in the context of liver cancer (16, 17). In addition, inhibitors of apoptosis, particularly inhibitors of BCL-2 family members or caspases, have recently been introduced to target several chronic inflammatory diseases including NASH. These inhibitors might not only prevent malignant transformation and, thus, be effective as preventive compounds, but also be viable therapeutic strategies for HCC. Together, inflammatory cell death is particular relevant for mechanistic and clinical applications in liver cancer.

The here presented review aims to summarize key cellular and molecular mechanisms involved in liver cell death during hepatocarcinogenesis with a main focus on apoptosis. We will also delineate the importance of predisposition to apoptosis as a key factor for malignant transformation and specify factors that affect differential predisposition to apoptotic stimuli during liver cancer development and therapy. Finally, the impact for personalized medicine and precision oncology will be discussed.

## Mechanisms of Cell Death in Hepatocarcinogenesis

Cell death is intrinsically associated with chronic inflammation in various organs including the liver (10). Herein, infectious and metabolic changes induced by the underlying etiological agent prone hepatocytes for further damage. Liver fibrogenesis and carcinogenesis are significantly accelerated by oxidative stress, cell death and inflammation. Thus, HCC is the final and most deadly consequence of all major chronic liver diseases (2). Consistently, continuous inflammatory cell death is one of the hallmarks of hepatocarcinogenesis. Almost all HCC patients show signs of cell death in sera and tissue and their emergence is indicative of adverse biological traits (18).

The apoptosis program governs the cell-autonomous removal of superfluous, infected, or damaged cells (19, 20) and thus constitutes the most prominent defense mechanism against hepatocarcinogenesis. During chronic damage, apoptosis is regulated on the outer mitochondrial membrane (OMM) by BCL-2 proteins. The pro-apoptotic BCL-2 proteins, BCL-2-associated X protein (BAX) and BCL-2 antagonist killer 1 (BAK) permeabilize the OMM and release intermembrane space proteins, such as cytochrome *c*, into the cytoplasm in order to activate the caspase cascade (21). Therefore, BAX and BAK can commit the cell to apoptosis. The cell is protected from BAX and BAK activity by functionally redundant pro-survival BCL-2 proteins. Although, BAX/BAK activation is usually followed by irreversible cellular commitment to apoptosis, cell survival is possible after limited OMM permeabilization (22). Even cells with the capacity to undergo death receptor-dependent apoptosis without mitochondrial apoptosis signaling enhance their apoptotic response by BAX/BAK activation (23). Therefore, therapeutic success of anti-tumor strategies, including targeted strategies, immune therapies as well as chemotoxic stress, rely on efficient BAX/BAK engagement in targeted cells. Several molecular alterations could be associated with induction or imbalance of pro- and anti-apoptotic BCL-2 proteins in liver cancer. They play an essential role in maintaining genomic integrity of hepatocytes. Disruption of the apoptotic program is frequently observed already during chronic liver diseases (12). Activation of BCL-x_L_ is further observed at high frequencies in human HCC, whereas concomitant downregulation of BAX is a common feature of HCC with p53 alterations and observed at progressed stages of the disease (24). Moreover, inhibition of caspases e.g., by XIAP is also common in human HCC and associated with TGFβ signaling and subsequent acquisition of pro-metastatic properties. In addition to the inhibition of pro-apoptotic proteins or caspases, activation of pro-survival genes as well as pathways contributes to liver cancer development and progression (25).

A prominent molecular alteration detected in a sizable number of HCC patients is NF-kB pathway that is also particularly important in metabolic liver diseases and NASH-induced HCC (26–28). The pathway controls diverse functions in a cell type and context-dependent manner and activity is observed during chronic inflammation, fibrogenesis as well as development and progression of HCC (29, 30). In hepatocytes, NF-κB mainly mediates survival during chronic damage in response to e.g., oxidative stress while suppression contributes to malignant transformation. However, NF-κB activation in non-parenchymal and immune cells can aggravate inflammation and fibrogenesis (31). Tumor necrosis factor-α and interleukin-6 are among the major inflammatory cytokines that induce this pathway. NF-κB downstream signaling resembling c-Jun N-terminal kinase (JNK), and signal transducer and activator of transcription 3 play a major role in inflammation-associated HCC (32). NF-κB activation can also be critically linked to several anti-apoptotic molecules including (cIAP1, cIAP2), XIAP, the BCL-2 family members A1 and BCL- x_L_, cFLIP, TRAF1, TRAF2, and GADD45β (33). Besides JNK, NF-κB also activates other pro-survival and pro-proliferative pathways, resembling p38 MAPK (mitogen-activated protein kinase) kinase (34, 35). In this context, upstream regulators, such as the NF-κB essential modulator (NEMO), the IKK kinase complex as well as death-domain kinase receptor-interacting protein kinase 1 (RIPK1) are of particular importance. The central regulators of cell death resembling TAK1 and RIPK1 are, consequently, other common findings mechanistically linked to malignant transformation in the liver. TAK1 (MAP3-kinase TGF-β-activated kinase 1) is critically involved in the modulation of innate and adaptive immune responses. Activation of TAK1 in parenchymal cells significantly inhibits apoptosis and demonstrated anti-tumorigenic effects mediated by NF-κB activation via TNF (36). Conversely, deficiency of TAK1 impaired NF-κB activity and induced hepatocyte apoptosis, inflammation as well as HCC development in a NEMO-dependent manner (36). Consistently, alteration of the immune cell composition and impairment of immune-mediated clearance of damaged hepatocytes is an important driver of liver cancer. It has recently been shown that dysregulation of lipid metabolism in NAFLD induces selective ablation of intrahepatic CD4+ cells, which impairs mitochondrial function and generates high levels of oxidative damage, thus, corroborating lipid dysregulation with impaired anti-tumor immune-surveillance (37). Accordingly, impaired senescence surveillance by myeloid cells also induced failure in immune-mediated clearance of damaged hepatocytes and accelerated hepatocarcinogenesis. ER stress induced by metabolic liver damage following a high fat diet further enhanced resulting liver damage, increased immune infiltration, and lipogenesis and, ultimately, led to HCC development (28).

Another form of cell death recently linked to HCC development is necroptosis. Again, the mentioned TAK1 model with liver-specific ablation was employed to clarify the relative contribution of necroptosis during hepatocarcinogenesis. While response to apoptosis in the model promoted inflammation and tumorigenesis, necroptotic response had opposing effects and conferred anti-inflammatory and tumor-suppressive functions. These results indicate the diverse molecular functions of key cell death pathways in mediating apoptotic, necroptotic or other forms of cell death. Detailed dissection of the relative contribution and mechanistic hallmarks are urgently needed (12).

An improved biological understanding of the exact mechanisms driving hepatocyte cell death and, ultimately, cancer growth are not only of particular scientific interest, but also directly imply translational applications. Besides identification of patients at risk for cancer development, biomarkers of cell death might also be instrumental to delineate the biological trait, i.e., prognosis, of a tumor but also for prediction and monitoring of treatment response. Nevertheless, excessive cell death was successfully identified predict the development as well as progression of liver cancer. Furthermore, expression of key markers in cell death and surrogate characteristics were associated with clinical outcome. As such, the new checkpoint molecules RIPK1 and TRAF2 were recently confirmed as independent prognostic markers in liver cancer (38). Furthermore, the serum cell death parameter M65, which detects cleaved and uncleaved CK-18 fragments, was also demonstrated to possess clinical utility as a non-invasive marker for tumor initiation as well as prognosis, corroborating the potential as a new diagnostic tool for HCC (16). Finally, it is well-established that transcriptome profiles conferring to cell death resistance are significantly enriched in HCCs with low differentiation, high invasion and a particularly poor outcome (39). In summary, imbalance of a broad range of molecules with critical function of cell death, including dysregulation of cytokines and inflammatory as well as survival pathways during chronic liver disease, possess high relevance for clinical application and harbor potential as translational biomarkers of malignant transformation as well as progression.

## Analysis of BCL-2 Proteins to Predict Tumor Cell Apoptosis

**Figure 1:**
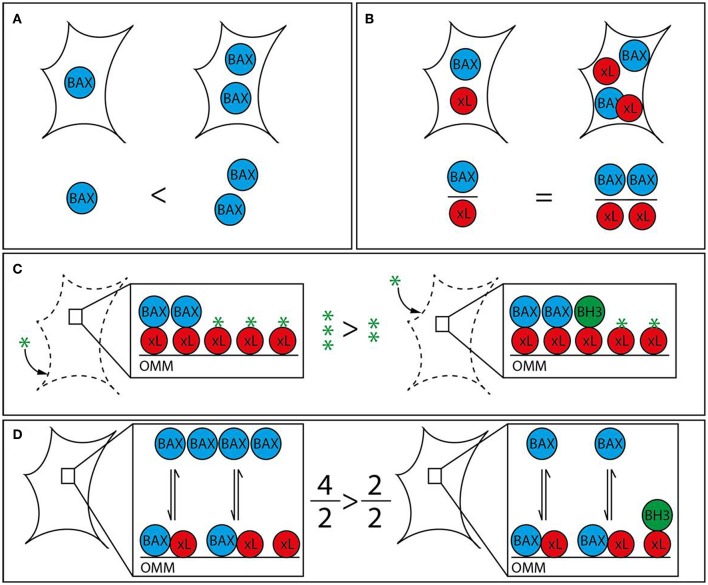
Strategies to analyze apoptotic predisposition based on BCL-2 proteins. **(A)** BAX level. The right cell contains more BAX (blue) than the left and is therefore considered to have a higher apoptosis predisposition. Other relevant factors are not measured. **(B)** BAX vs. BCL-xL level. The ratio between BAX and a single pro-survival BCL-2 protein (BCL-xL, red) is similar in both cells. Therefore, both cells would be judged to have the same tendency to initiate apoptosis. The redundancy of the BCL-2 family would require this analysis to be expanded to all BCL-2 proteins in order to be insightful. **(C)** BH3 profiling. Permeabilized and cultured cells are incubated with BH3 peptides (green star) in order to titrate the amount of free BH3 binding sites on the outer mitochondrial membrane (OMM). BH3-only proteins (green) associated with the OMM following prior cell stress reduce the amount of free BH3 binding sites and thus increase the sensitivity toward BH3 mimetics. The increased capacity of the left cell would translate into a reduced sensitivity toward BH3 mimetics. **(D)** Relative BAX localization. Determination of the cytosolic and mitochondrial BAX pools in intact cells describes the position of the BAX localization equilibrium and thus the cellular predisposition to apoptosis. While single contributing factors cannot be dissected, all contributing factors, e.g., BCL-2 protein interactions with BH3 motifs and other segments, interacting proteins outside the BCL-2 family, are included. BH3-only proteins reduce the shuttling rate and thus the cytosolic BAX pool. The larger cytosolic pool of the left cell shows reduced predisposition to apoptosis. The analysis can be supplemented with measuring the functionally redundant and similarly regulated BAK, which is usually shifted toward the mitochondria but shows a similar range of localizations in human samples.

While liver tumors possess molecular characteristics that set them apart from other types of tumors, general mechanisms of apoptosis regulation apply as they have been shown in many different cell types. The discovery of opposing BCL-2 protein activities led to the rheostat model to describe regulatory interactions in mitochondrial apoptosis signaling (40, 41). The model postulates that pro-survival BCL-2 proteins act anti-apoptotic by binding to BAX and BAK. Therefore, mitochondrial apoptosis would largely dependent on different protein expression and degradation rates. In fact, platelets contain a molecular timer that commits them to apoptosis when BAK levels exceed the levels of the predominant pro-survival BCL-2 (42). The rheostat model sparked a body of work suggesting prediction of therapeutic success based on measuring BAX levels (Figure 1). The refined version of this approach investigated the BAX/BCL-2 ratio. However, subsequent research expanded our knowledge on protein localizations and interactions, revealing the absence of the prerequisite of the rheostat model: stable protein complexes. Prediction of apoptotic outcome based on protein levels encountered another major problem with the discovery of new members of the BCL-2 family. Their functional redundancies forsake all educated guesses, whether pro-survival BCL-2 proteins outnumber pro-apoptotic BCL-2 proteins. Therefore, apoptosis predictions based on protein levels, although occasionally attempted, are unrewarding.

**Figure 2:**
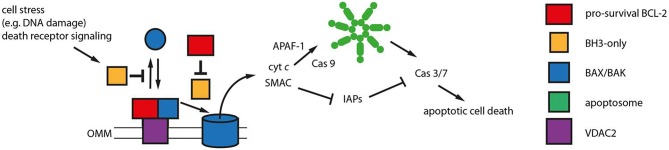
Mitochondrial apoptosis signaling. Mitochondrial apoptosis is regulated by members of the BCL-2 protein family on the outer mitochondrial membrane (OMM). The pro-apoptotic BCL-2 proteins BAX and BAK (blue) constantly translocate to the OMM undergoing a conformational change. The porin voltage-dependent anion channel 2 (VDAC 2, purple) acts as mitochondrial BAX/BAK receptor and as platform for the retrotranslocation of BAX and BAK back into the cytosol dependent on the activities of pro-survival BCL-2 proteins (red). The equilibrium between BAX/BAK translocation and retrotranslocation determines the cellular predisposition to apoptosis. Intrinsic stress as well as death receptor signaling is mediated by BH3-only proteins (yellow) that inhibit BAX/BAK retrotranslocation shifting BAX and BAK toward the mitochondria. The BH3-only proteins tBID, BIM and PUMA are also thought to directly activate BAX and BAK initiating OMM permeabilization and the release of cytochrome *c* (cyt *c*) and SMAC into the cytosol. This function can be inhibited by pro-survival BCL-2 proteins. Cytosolic cyt *c* initiates in turn the formation of the apoptosome (green), an APAF-1 complex activating Caspase 9 (Cas 9). Subsequently, Caspases 3 and 7 are activated that can be inhibited by IAPs in the absence of SMAC in the cytosol. Caspase 3/7 activation leads to the efficient dismantling of the cell into apoptotic bodies that are later phagocytosed.

A group of proteins that has influenced revised paradigms for apoptosis signaling and predictions of apoptotic outcome contains BH3-only proteins (Figure 2). BH3-only proteins are defined by harboring a single BH3 motif, while the remaining protein structures diverge as much as the type of stress signaled to the OMM, including DNA damage, ER stress, death receptor signaling and other types of stress (43). BH3-only proteins are thought to either inhibit pro-survival BCL-2 proteins and/or directly activate BAX and BAK (44). Inhibition of pro-survival BCL-2 proteins by BH3-only proteins is structurally well-characterized and has led to the development of low molecular weight inhibitors. These targeted anti-cancer small molecule inhibitors called BH3 mimetics bind to and inhibit pro-survival BCL-2 proteins in a manner similar to BH3-only proteins.

The concept that BH3-only proteins loaded on the OMM could determine the cellular response to apoptosis has led to the strategy to profile BH3-only proteins. “Mitochondrial priming” in this context is the resulting stress capacity of cells dependent on the presence of pro-survival BCL-2 proteins, OMM-accumulated BH3-only proteins and BAX/BAK (Figure 1). Actually, BH3 profiling is again based on the rheostat model. It expands the model by emphasizing the potential role of BH3-only proteins, but does not take into account the transient nature of BCL-2 protein interactions and interactions among BCL-2 proteins other than through the BH3 motif. Extensive work shows the feasibility of “BH3 profiling” in different cellular settings (45–49). The analysis involves the short culturing of cells, limited cell lysis, incubation with peptides corresponding to BH3 domains and the analysis of OMM permeabilization through a membrane potential-sensitive dye. Cell culturing is prone to changes the apoptotic predisposition of a given tumor clone despite relative genetic stability. In addition, recent research has provided evidence of several secondary binding sites in BCL-2 protein interactions that BH3 profiling cannot account for (50–53). Therefore, BH3 profiling can particularly identify the contribution of pro-survival BCL-2 activities to the survival and therefore support selection of the potentially most effective BH3 mimetic. On the other hand, the appropriate BH3 mimetics could be tested directly, as procedure and readout would be similar.

## Apoptosis Predisposition by the Position of the BAX/BAK Localization Equilibrium

Prior stress and stress response influence the apoptotic predisposition but are also reflected in the cellular localization of the pro-apoptotic BCL-2 proteins. Despite their functional redundancy, BAK is found largely on the OMM in many cell types, while BAX resides primarily in the cytoplasm (54, 55). This apparent difference is important, since the sizes of the mitochondrial protein pools prior apoptotic stress determines apoptotic response (56). The mitochondrial BAX pool as much as the corresponding BAK pool is variable because both proteins are inhibited by a dynamic shuttling equilibrium between cytosol and mitochondria (57). Pro-survival BCL-2 proteins constantly retrotranslocate BAX and BAK from the mitochondria and cell stress mediated by BH3-only proteins shifts both pro-apoptotic BCL-2 proteins back onto the mitochondria. The importance of mitochondrial BAX for apoptosis induction implies that (i) the total cell protein population is not critical for apoptosis induction and (ii) accurately measuring mitochondrial BAX (or BAK) fractions or shuttling rates could predict apoptotic outcome in response to stress (Figure 1). Experimental observations have shown that the ratio between cytosolic and mitochondrial BAX/BAK is the best available representation of the average localization dynamics of BAX/BAK molecules (58). The paradigm that relevant protein pool and total protein level are not necessarily connected is true for BAX, BAK, pro-survival proteins, like BCL-2 and BCL-x_L_, and BH3-only proteins, like BID (53, 59, 60). Relative BAX/BAK localization reflects the combined contributions of all players, known and unknown, to the cellular predisposition to apoptosis. Similar differences in the cellular BAX localization could also be present in HCC and could be associated with distinct molecular and clinical characteristics of the tumors.

## Targeting of Cell Death as a Therapeutic Strategy for HCC

Hepatocyte damage and consecutive activation of cell death signaling plays a pivotal role for liver cancer initiation, but is also of particular importance for modulating treatment effects during established therapies. Herein, cell death can be induced by chemotherapeutic as well as targeted approaches (61). Sorafenib and lenvatinib are the only approved first line therapies for advanced stages in liver cancer (62). Both compounds are multi-tyrosine kinase inhibitors with anti-angiogenic properties. It is well-known, that sorafenib is a strong inducer of apoptosis and exposure to hepatoma cells leads to BAX/BAK activation, at least in part through the BH3-only protein PUMA (63). Furthermore, high numbers of objective response rates observed in HCC patients further indicate that considerable cell death follows lenvatinib treatment (64). However, several recent reports suggest that in addition to induction of cell death the treatment effect is also significantly induced by immunomodulation through targeted therapies (65). In consequence, several combination therapies with PD1/PD-L1 therapies are currently under clinical evaluation (62).

Direct targeting of cell death pathways and modulation of the apoptotic response might be a viable preventive strategy in chronic liver diseases but also exert direct anti-tumorigenic properties in HCC (Table 1). Given central role of p53 as a regulator of cell death, restoration of its function was attempted to induce anti-tumor activity in several studies. While adenoviral delivery of recombinant p53 did not reveal promising results, modulation of p53 activity by e.g., ubiquitination through inhibition of COP1, was recently explored (66). Blockade of COP1 by systemic delivery of RNAi decreased *in vivo* cancer growth and significantly induced apoptosis in several HCC cell lines. Furthermore, several compounds were identified to restore p53 functions. Prominently, p53 reactivation and induction of massive apoptosis (PRIMA-1) and PRIMA-1Met are currently evaluated in several clinical trials (67). In the liver, application of the compounds is currently restricted to preclinical data and shows promising anti-tumor effects when mutant p53 is silenced by siRNA. Other therapeutic strategies aimed to directly target proteins involved in apoptosis to enhance the apoptotic response of cancer therapies. Interestingly, XIAP antisense therapy in combination with sorafenib showed synergistic anti-tumor effects in a recent phase II clinical trial (68). Results showed a moderate increase in progression-free survival (4.0 months vs. 2.6 months), overall survival (6.5 months vs. 5.4 months), and objective response rates (16.1% vs. 9.7%) compared with Sorafenib monotherapy. Notably, drug-related adverse events were moderate.

**Table 1:** Selected targets of cell death in liver diseases and cancer.

**Drug**	**Target, function, pathway**	**Target population**	**Phase clinical development**
PRIMA-1	Restoration of p53 function	Pre-clinical	N/A
Emricasan/IDN-6556 GS9450	Pan-Caspase Inhibitor	NASH, liver cirrhosis	Phase II (e.g., NCT02960204, NCT02686762, NCT03205345)
Venetoclax/ABT-199	BH3 mimetic	Pre-clinical	N/A
GSK2982772	RIPK1/RIPK3 Inhibitor	Pre-clinical	N/A
Etanercept	TNF Inhibitor	Alcoholic hepatitis, chronic viral hepatitis, NAFLD/NASH, AIH, PBC	Phase I-II
AEG35156	XIAP Antisense	HCC	Phase I-II (e.g., NCT00882869)
Curcumin	NF-kB, RIPK Inhibitor	HCC, NAFLD/NASH	Phase I-II (e.g., NCT03864783)

Pro-survival BCL-2 proteins are also under intensive preclinical and clinical evaluation as cancer therapy targets. The use of the BH3 mimetic venetoclax or ABT-199 in chronic lymphocytic leukemia (CLL) has shown the potential of this strategy, as response rates of about 80% can be achieved with single-agent venetoclax even in a relapsed/refractory setting (69). Current efforts explore the combination of venetoclax with rituximab, obinutuzumab or ibrutinib in order to suppress acquired resistance observed during monotherapy (70, 71). Venetoclax in combination with hypomethylating agents (HMAs) has also received special attention for the treatment of acute myeloid leukemia (AML) (72, 73). The combination has been shown to target leukemia stem cells (74). In the liver, recent evidence suggest that BH3-only protein BID significantly contributes to the development of liver cancer (75). Loss of BID was shown to delay hepatocarcinogenesis by reducing cell death, liver inflammation, and compensatory proliferation (76). Thus, modulation of the BCL-2 protein interplay might be a promising therapeutic strategie for liver cancer.

In addition to the therapeutic targeting of critical apoptosis regulators, pan-caspase inhibitors, e.g., Emricasan/IDN-6556, or selective caspase-1,8,9 inhibitors, e.g., GS9450, have been explored in preclinical models as well as clinical trials, mainly in the context of chronic liver diseases (77). While the majority of these trials showed improved liver enzymes as a surrogate for hepatocyte protection, effect on degree of hepatitis and fibrogenesis is still unclear and is currently under evaluation in large phase III trials for the treatment NASH with and without liver cirrhosis (NCT02960204, NCT02686762, NCT03205345). Importantly, caspase inhibition might induce necroptosis or other complications and, thus, require further investigations addressing the safety of long-term administration (12).

Although no clinical trials have yet been initiated to test the clinical efficacy of necroptosis inhibition in liver disease, preclinical studies and early phase clinical trials in inflammatory (auto-immune) disease indicate that inhibition of RIPK1 kinase activity might also be a promising therapeutic strategy and prevent apoptosis in chronic liver diseases (78). However, while the importance of several key proteins including RIPK1, TAK1, and NEMO has been shown, the therapeutic potential for HCC remains to be demonstrated. Based on the regulatory functions of RIPK1, inhibition might even cause paradox reactions depending on the context of inhibition and affected cell type (30). Finally, given the redundancy in the different pathways, combination of different anti-tumor therapies with one or several modulators of cell death pathways might be of particular therapeutic potential.

## Summary and Conclusions

Hepatocyte death is a key driver of chronic inflammatory liver diseases and hepatocarcinogenesis. Several lines of evidence suggest that apoptosis and other types of cell death are critically linked to initiation and progression of liver cancer. They participate in shaping the biological trait of the tumor, thus, ultimately determining patient prognosis. Herein, existence and degree of cell death infers several mechanistic and translational implications. While detection of the apoptotic predisposition might be a powerful diagnostic tool, direct targeting of mitochondrial apoptosis might complement the limited therapeutic strategies for HCC. In light of recent advances in immune-oncological approaches, targeting of cell death might also exert synergistic immuno-modulatory properties that could be explored in combination treatment strategies. However, our understanding of the detailed mechanisms and triggers of activation underlying the diverse mechanisms of cell death remains limited. Thus, definition of the actual state of cell death signaling effect in the distinct parenchymal and non-parenchymal cell types within the liver is urgently needed. Furthermore, the relative importance for distinct disease stages, i.e., chronic liver disease, tumor initiation as well as progression should be conclusively dissected to advance the field and before application of specific modulators of cell death in human is warranted. Furthermore, intensive translational research is needed to characterize the molecular hallmarks that operate on the intersection between cell death and inflammation. In this context, individual predisposition to apoptosis of cancer cells or cells within the hepatic microenvironment might be of particular relevance and might require distinct therapeutic strategy, thus, precision oncological approaches. Nevertheless, targeting of apoptosis is a promising avenue of HCC treatment that might yield to novel treatment strategies for this deadly inflammatory-driven cancer.

## Author Contributions

JM and FE conceptualized, wrote, and edited the review.

### Conflict of Interest

The authors declare that the research was conducted in the absence of any commercial or financial relationships that could be construed as a potential conflict of interest.
